# Socioeconomic determinants of leprosy in Brazilian municipalities: an ecological study^؆^

**DOI:** 10.1016/j.abd.2026.501396

**Published:** 2026-06-16

**Authors:** Aroldo de Oliveira Gomes Golineli, Maria Cecília Valsechi Belli, Guilherme Henrique Lourenço, Olympio Belli, Hélio Amante Miot

**Affiliations:** a Department of Infectology, Dermatology, Imaging Diagnosis and Radiotherapy, Faculty of Medicine, Universidade Estadual Paulista, Botucatu, SP, Brazil; b Faculty of Mechanical Engineering, Universidade Estadual de Campinas, Campinas, SP, Brazil

Dear Editor,

Leprosy is a neglected disease strongly influenced by socioeconomic vulnerability. Although Brazil achieved a decline in the detection of new cases, leprosy remains endemic and unevenly distributed across its territory.^1^ The number of newly diagnosed cases declined by 28.7%, from 31,064 in 2014 to 22,129 in 2024; however, transmission remains spatially clustered, with marked spatial dependence persisting across municipalities (Fig. 1).

**Figure 1:**
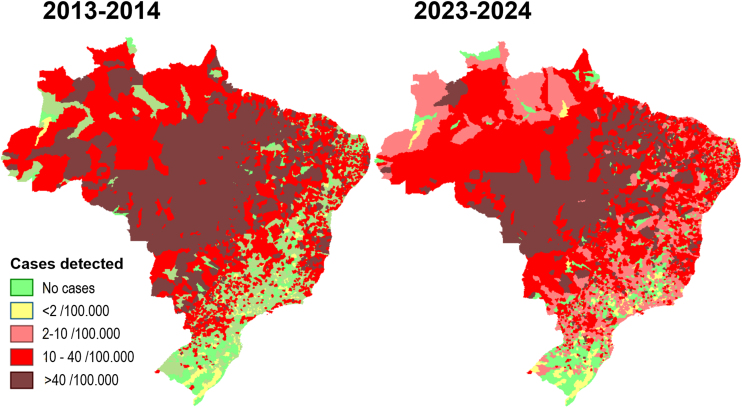
Spatial distribution of case detection of leprosy in Brazilian municipalities. Pooled data from 2013‒2014 and 2023‒2024.

Socioeconomic determinants modulate infectious disease dynamics by influencing exposure, susceptibility, and access to timely diagnosis and treatment. Structural factors such as poverty, education, income distribution, housing conditions, and sanitation critically affect the persistence of leprosy, particularly in regions marked by inequality and restricted access to healthcare.^2^, ^3^, ^4^, ^5^

While individual-level studies have demonstrated strong associations between deprivation and leprosy, fewer analyses have explored how multidimensional social progress and municipal development indicators jointly shape population-level leprosy behavior across Brazil.^5^, ^6^ This ecological study examined epidemiological characteristics of leprosy in Brazil regarding demographic, social, economic, and geographic determinants.

We analyzed publicly available municipal data from Brazilian national databases, including leprosy notifications (https://indicadoreshanseniase.aids.gov.br/), indicators from the 2022 Brazilian Census (https://censo2022.ibge.gov.br/), Human Development Index (HDI 2025; www.atlasbrasil.org.br), Gini coefficient 2010 (www.data.worldbank.org), Social Progress Index (SPI 2024; https://ipsbrasil.org.br/), and dermatologists registered in the Brazilian Society of Dermatology (SBD ‒ www.sbd.org.br).

Primary outcomes were the municipal incidence of leprosy, pooled for 2013‒2014 and 2023‒2024, and its variation across the period. Municipalities with non-zero incidence were included in each analysis. Independent variables comprised latitude, longitude, mean residents per household, degree of urbanization, demographic density, Gross Domestic Product (GDP) per capita, ethnic composition, dermatologist density, HDI, SPI, and Gini coefficient. Correlations were assessed using Spearman’s ρ. A generalized modeling approach was applied with a probability distribution defined according to each outcome (linear or ordinal logit). Collinearity was evaluated by the Variance Inflation Factor (VIF), and autocorrelation by Durbin-Watson. Significance was set at p ≤ 0.001 to control false positives due to multiple correlations.

Of 5,570 municipalities, 3,987 (71.6%) reported new leprosy cases in 2013‒2014 and 3,715 (66.7%) in 2023–2024. Fig. 2 shows bivariate correlations between incidence in both periods, incidence variation, and covariables, emphasizing the relationships among the studied variables.

**Figure 2:**
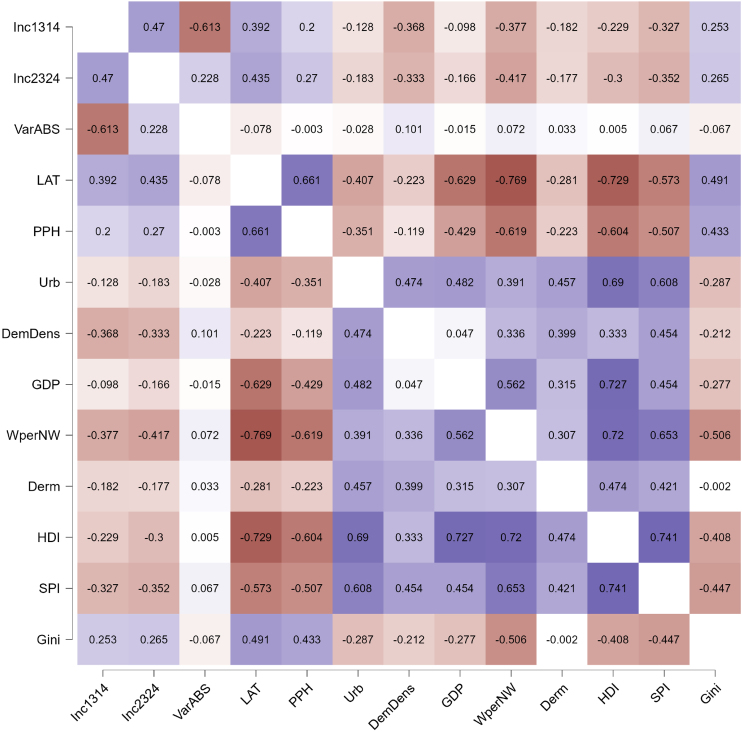
Heatmap of Spearman correlation coefficients among leprosy indicators and municipal-level determinants. Spearman’s ρ coefficients illustrating correlations between mean incidence of new leprosy cases in 2013–2014 (Inc1314) and 2023–2024 (Inc2324), absolute reduction in incidence between periods (VarABS), and demographic, social, economic, and geographic covariables: Latitude (LAT), People Per Household (PPH), Urban population proportion (Urb), Gross Domestic Product Per capita (GDP), ethnicity distribution (White, Black, Brown, Yellow, Amerindian), Dermatologist Density (Derm), Human Development Index (HDI), Social Progress Index (SPI), Demographic Density (DemDens), and Gini coefficient (n = 4,467). Blue, Negative association; Red, Positive association.

Table 1 summarizes factors associated with the mean incidence in 2023‒2024. Higher municipality latitude, higher proportion of admixed ethnicity, increased household crowding, and greater dermatologist density, along with lower demographic density and reduced social progress, were noteworthy.

**Table 1:** Demographic, geographic, economic, and social determinants associated with the (log-transformed) mean incidence of new leprosy cases in Brazilian municipalities, 2023–2024 (n = 3,715). Univariate (β) and multivariate (standardized β) regression coefficients, Standard Errors (SE), and p-values for municipal-level predictors of leprosy incidence.

Variables	β^a^	SE^a^	p-value^a^	β_SE_^b^	p-value^b^
Latitude (degrees)	0.028	0.001	<0.001	0.343	<0.001
Longitude (degrees)	-0.007	0.001	<0.001	‒c	‒
People per household	0.241	0.024	<0.001	0.166	<0.001
Urban population (%)	-0.677	0.040	<0.001	-0.086	<0.001
Demographic density (1000 persons/Km^2^)	-0.111	0.009	<0.001	-0.138	<0.001
Gross Domestic Product per capita (R$1000/year)	-0.001	0.000	<0.001	-0.081	<0.001
Self-declared white (%)	-0.011	0.000	<0.001	‒c	‒
Self-declared black (%)	0.008	0.001	<0.001	‒c	‒
Self-declared brown (%)	0.013	0.000	<0.001	0.242	<0.001
Self-declared yellow (%)	-0.164	0.019	<0.001	‒c	‒
Self-declared Amerindian (%)	0.004	0.001	0.006	‒c	‒
Dermatologists (per 100,000 inhabitants)	-0.047	0.003	<0.001	-0.097	<0.001
Human Development Index	-2.257	0.105	<0.001	0.286	<0.001
Social Progress Index	-0.038	0.001	<0.001	-0.299	<0.001
Gini coefficient	1.351	0.132	<0.001	0.050	0.003

p (model) < 0.001, p (constant) < 0.001; R^2^ (standardized) = 30.7%; Durbin-Watson: 1.60.

a Bivariate analysis.

b Multivariate analysis.

c Variable excluded due to collinearity (VIF > 5).

When analyzed separately, components of HDI were associated with leprosy incidence: HDI longevity (βSE = -0.14), HDI education (βSE = -0.31), and HDI income (βSE = -0.33). Moreover, among the components of SPI (Supplemental Material Table S1), incidences of leprosy were more strongly associated with environmental quality (βSE = -0.50), foundations of wellbeing (βSE = -0.48), fire hotspots (βSE = 0.28), and adolescent pregnancy (βSE = 0.40).

Table 2 shows determinants associated with changes in incidence between 2013‒2014 and 2023‒2024. Municipalities with higher HDI, a more urbanized population, and a higher baseline incidence presented a greater probability of reduction in case detection over the period. When analyzed separately, all the components of HDI contributed to the leprosy reduction: HDI longevity (OR = 0.26), HDI income (OR = 0.34), and HDI education (OR = 0.51).

**Table 2:** Municipal determinants associated with the variation in new leprosy diagnoses between 2013–2014 and 2023–2024 in Brazil (reduction, stability, or increase). Ordinal regression model showing Odds Ratios (OR), 95% Confidence Intervals (95% CI), and p-values for factors associated with municipal change in leprosy incidence across the decade. Variables with p ≤ 0.01 in the bivariate analysis were retained in the multivariate model (n = 4,467).

Variables	OR (95% CI)^a^	p-value^a^	OR (95% CI)^b^	p-value^b^
Latitude (degrees)	1.00 (0.99‒1.01)	0.433	‒	‒
Longitude (degrees)	1.03 (1.02‒1.04)	<0.001	1.00 (0.98‒1.00)	0.992
People per household	0.93 (0.78‒1.10)	0.387	‒	‒
Urban population (%)	0.52 (0.39‒0.69)	<0.001	0.59 (0.39‒0.90)	<0.001
Demographic density (1000 persons/km^2^)	1.00 (1.00‒1.00)	0.700	‒	‒
Gross Domestic Product per capita (R$1000/year)	1.00 (0.99‒1.01)	0.459	‒	‒
Self-declared white (%)	1.00 (0.99‒1.01)	0.150	‒	‒
Self-declared black (%)	1.00 (0.99‒1.02)	0.096	‒	‒
Self-declared brown (%)	1.00 (0.99‒1.00)	0.044	‒	‒
Self-declared yellow (%)	0.83 (0.72‒0.96)	0.012	‒	‒
Self-declared Amerindian (%)	1.00 (0.99‒1.01)	0.556	‒	‒
Dermatologists (per 100,000 inhabitants)	1.00 (0.99‒1.02)	0.574	‒	‒
Human Development Index	0.36 (0.17‒0.76)	0.007	0.06 (0.02‒0.21)	<0.001
Social Progress Index	1.00 (0.99‒1.02)	0.456	‒	‒
Gini coefficient	0.27 (0.11‒0.65)	0.004	0.63 (0.23‒1.77)	0.381
Incidence in 2013‒2014 (cases / 100.000)	0.97 (0.96‒0.98)	<0.001	0.97 (0.96‒0.98)	<0.001

p (model) < 0.001; OR (95% CI), Odds Ratio (95% Confidence Interval).

a Bivariate analysis.

b Multivariate analysis.

Our findings are consistent with previous evidence indicating that socioeconomic vulnerability plays a role in leprosy persistence in Brazil.^3^, ^7^ Poverty, low education, poor housing conditions, and household crowding significantly increase the risk of leprosy in highly endemic countries, reinforcing the role of structural deprivation in sustaining transmission.^4^ Similarly, in a Brazilian Cohort, individuals living in socially deprived settings have a higher probability of being diagnosed with leprosy, emphasizing that risk is deeply embedded in social stratification.^5^ Municipalities with indicators of social vulnerability displayed higher municipal-level incidence and reduced decline over time. However, unlike individual-level designs, our results highlight how these determinants operate collectively, shaping municipal epidemiological behavior. In addition, access to qualified dermatologic care may also modulate disease burden, possibly increasing diagnostic accuracy. Altogether, our results suggest that effective leprosy control in Brazil depends not only on biomedical strategies, but also on sustained social development policies and equitable strengthening of healthcare networks.

Historically, Europe experienced a marked decline in leprosy between the Middle Ages and the 19^th^ century in the absence of effective antimicrobial treatment, largely attributed to improvements in nutrition, housing, sanitation, and overall living standards.^8^ Similar structural determinants remain unresolved in several Brazilian regions, where poverty, household crowding, and sanitation vulnerability contribute to continued transmission and delayed diagnosis.

Environmental issues, lower education, income, teenage pregnancies, ethnic admixture, and high latitudes emerged as associated with a higher incidence of leprosy. In 2024, the North, Northeast, and Midwest regions of Brazil accounted for 80.5% of the new diagnoses. Despite no proven climate-only causality hypothesis, the current endemic countries are situated in the intertropical areas of the Americas, Africa, Asia, and Oceania.

Higher longevity was the HDI component associated with greater disease reduction between the periods, reflecting the aggregate role of different elements. Better healthcare, food safety, sanitation, public safety, and social well-being contribute to a simultaneous increase in longevity and capacity to deal with endemic situations.^9^

Leprosy remains diagnostically complex, given its long incubation period and heterogeneous cutaneous and neurologic manifestations, which pose challenges to primary healthcare services. In Brazil, the distribution of board-certified dermatologists is uneven and largely concentrated in metropolitan areas.^10^ Municipalities with greater dermatologist availability showed lower incidence of leprosy, which may reflect both improved diagnostic capability and the broader municipal health infrastructure. However, the progressive reduction of specialized outpatient clinics dedicated to leprosy care across the country must be considered when assessing diagnostic capacity. Additionally, the training of other medical specialties (and non-medical health professionals) in the recognition of leprosy, as well as shifts in dermatology training and clinical practice patterns, should be taken into account, since accurate diagnosis requires solid expertise in dermatoneurological examination.

This study has limitations inherent to ecological designs, the quality of notification data across the municipalities, and some differences in the temporal availability of socioeconomic indicators. The non-inclusion of municipalities with zero incidence does not necessarily indicate the absence of cases and may instead reflect underdiagnosis. However, these constraints do not invalidate the consistency and plausibility of the associations identified. Future studies should integrate spatial analysis with individual and household data to clarify how municipal-level determinants shape personal risk, exposure, diagnosis, and disability in leprosy. Finally, the impact of the COVID-19 pandemic may still be influencing the epidemiological indicators for 2023/24.^1^, ^11^

In conclusion, the distribution of leprosy in Brazil is shaped by demographic, geographic, and social determinants. Policies aimed at reducing vulnerability, improving social progress, strengthening primary care, and expanding equitable access to specialized dermatologic services are crucial for advancing disease control and reducing the disability burden.

## ORCID IDs

Aroldo de Oliveira Gomes Golineli: 0009-0007-5267-7178

Maria Cecília Valsechi Belli: 0009-0002-4003-1664

Guilherme Henrique Lourenço: 0009-0004-5283-2957

Olympio Belli: 0000-0001-9362-8507

## Declaration of Generative AI and AI-assisted technologies in the writing process

During the preparation of this work, the authors used ChatGPT 5.0 to assist with English language editing. After using this tool, the authors reviewed and edited the content as needed and take full responsibility for the content of the published article.

## Research data availability

The entire dataset supporting the results of this study was published in this article.

## Financial support

CNPq (306358/2022-0) – Hélio Amante Miot is a CNPq researcher.

## Authors' contributions

Aroldo de Oliveira Gomes Golineli: Conception and study design; data collection and interpretation; manuscript drafting; critical revision of the manuscript for important intellectual content; critical review of the literature; approval of the final version of the manuscript.

Maria Cecília Valsechi Belli: Conception and study design; data collection and interpretation; manuscript drafting; critical revision of the manuscript for important intellectual content; critical review of the literature; approval of the final version of the manuscript.

Olympio Belli: Data collection and interpretation; critical revision of the manuscript for important intellectual content; critical review of the literature; approval of the final version of the manuscript.

Guilherme Lourenço: Conception and study design; data collection and interpretation; manuscript drafting; critical revision of the manuscript for important intellectual content; critical review of the literature; approval of the final version of the manuscript.

Hélio Amante Miot: Conception and study design; data collection, analysis, and interpretation; statistical analysis; manuscript drafting; critical revision of the manuscript for important intellectual content; critical review of the literature; approval of the final version of the manuscript.

## Conflicts of interest

None declared.
