# Supplementary material for: Socioeconomic determinants of leprosy in Brazilian municipalities: an ecological study
Source: An Bras Dermatol. 2026 Jun 16;101(4):501396. doi: 10.1016/j.abd.2026.501396 (PMC13292794; doi:10.1016/j.abd.2026.501396)
Supplement: Supplementary file 1 [file mmc1.docx]

ABD-D-25-00956_Supplementary Material

**Supplementary Material** Correlation between the pooled (2023‒2024) incidence of leprosy in 3,715 Brazilian municipalities and the items from the Social Progress Index.

| **Social Progress Index ‒ Item** | **Spearman’s rho** |
| --- | --- |
| Basic Human Needs | -0.33 |
| Foundations of Wellbeing | -0.46 |
| Opportunities | -0.11 |
| Nutrition and Basic Medical Care | -0.12 |
| Water and Sanitation | -0.30 |
| Housing | -0.22 |
| Personal Safety | -0.13 |
| Access to Basic Knowledge | -0.21 |
| Access to Information and Communication | -0.39 |
| Health and Wellness | -0.06 |
| Environmental Quality | -0.48 |
| Individual Rights | 0.01 |
| Personal Freedom and Choice | -0.40 |
| Social Inclusion | 0.24 |
| Access to Higher Education | -0.24 |
| Vaccination Coverage (Poliomyelitis) | 0.02 |
| Hospitalizations for Primary Care-Sensitive Conditions | -0.01 |
| Mortality Adjusted for Primary Care-Sensitive Conditions | 0.01 |
| Under-5 Mortality | 0.16 |
| Undernutrition | 0.15 |
| Water Supply via Public Distribution Network | -0.26 |
| Adequate Sanitary Sewage | -0.32 |
| Water Supply Index | -0.20 |
| Water Distribution Loss Index | 0.03 |
| Households with Adequate Waste Collection | -0.30 |
| Households with Adequate Electric Lighting | 0.13 |
| Households with Adequate Walls | -0.27 |
| Households with Adequate Flooring | -0.22 |
| Youth Homicides | 0.12 |
| Female Homicides | 0.02 |
| Deaths from Traffic Accidents | 0.19 |
| Homicides | 0.22 |
| Dropout in Primary School | 0.12 |
| Dropout in Secondary School | 0.05 |
| Secondary School Truancy | 0.16 |
| Age–Grade Distortion in Secondary School | 0.17 |
| Primary Education IDEB Index | -0.31 |
| Secondary School Failure Rate | -0.07 |
| Mobile Internet Coverage (4G/5G) | -0.39 |
| Fixed Broadband Internet Density | -0.38 |
| Mobile Phone Density | -0.23 |
| Mobile Internet Quality | -0.15 |
| Life Expectancy | -0.31 |
| Mortality Between Ages 15–50 | 0.15 |
| Mortality from Non-Communicable Chronic Diseases | -0.09 |
| Obesity | -0.27 |
| Suicides | -0.02 |
| Urban Green Areas | -0.25 |
| CO₂ Emissions per Inhabitant | 0.34 |
| Heat Foci (Burning/Fire Hotspots) | 0.44 |
| Municipal Climate Vulnerability Index | 0.25 |
| Suppression of Primary and Secondary Vegetation | 0.33 |
| Access to Human Rights Programs | -0.04 |
| Existence of Minority Rights Actions | -0.06 |
| Justice System Demand Response Index | 0.08 |
| Court Case Backlog Rate | -0.12 |
| Access to Culture, Leisure, and Sports | -0.36 |
| Adolescent Pregnancy (< 19-years) | 0.42 |
| Urban Squares and Parks | -0.13 |
| Child Labor | 0.15 |
| Gender Parity in Municipal Legislature | 0.09 |
| Black Representation Parity in Municipal Legislature | 0.12 |
| Violence Against Indigenous People | -0.18 |
| Violence Against Women | -0.22 |
| Violence Against Black People | -0.18 |
| Workers with Higher Education | -0.18 |
| Women Employed with Higher Education | -0.15 |
| Average ENEM Score | -0.40 |
